# Low serum phosphate as an independent predictor of increased infection-related mortality in dialysis patients: A prospective multicenter cohort study

**DOI:** 10.1371/journal.pone.0185853

**Published:** 2017-10-03

**Authors:** Ji-Eun Lee, Jeong-Hoon Lim, Hye Min Jang, Yon Su Kim, Shin-Wook Kang, Chul Woo Yang, Nam-Ho Kim, Eugene Kwon, Hyun-Ji Kim, Jeung-Min Park, Hee-Yeon Jung, Ji-Young Choi, Sun-Hee Park, Chan-Duck Kim, Jang-Hee Cho, Yong-Lim Kim, for the Clinical Research Center for End Stage Renal Disease Investigators

**Affiliations:** 1 Department of Internal Medicine, Kyungpook National University School of Medicine, Daegu, Korea; 2 Clinical Research Center for End Stage Renal Disease, Daegu, Korea; 3 Department of Statistics, Kyungpook National University, Daegu, Korea; 4 Department of Internal Medicine, Seoul National University College of Medicine, Seoul, Korea; 5 Department of Internal Medicine, Yonsei University College of Medicine, Seoul, Korea; 6 Department of Internal Medicine, Catholic University of Korea College of Medicine, Seoul, Korea; 7 Department of Internal Medicine, Chonnam National University Medical School, Gwangju, Korea; Hospital Universitario de la Princesa, SPAIN

## Abstract

**Background:**

The role of mineral metabolism in mortality among dialysis patients has received increased attention, but some aspects remain unclear. The aim of the present study was to investigate the prognostic value of serum calcium and phosphate levels for all-cause mortality and cause-specific mortality in dialysis patients.

**Methods:**

Patients on hemodialysis and peritoneal dialysis were enrolled from a multicenter prospective cohort study in Korea (NCT00931970). The patients were divided into low, normal, and high groups according to their baseline serum calcium or phosphate levels. Cox proportional analysis and a proportional hazards model for the subdistribution of a competing risk were used to calculate hazard ratios (HRs) for the association of serum calcium and phosphate levels with all-cause and cause-specific mortality. Time-dependent values of calcium and phosphate were also evaluated to assess the effect of longitudinal change in mineral metabolism parameters on mortality types.

**Results:**

A total of 3,226 dialysis patients were followed up for a mean of 19.8 ± 8.2 months. Infection was the most common cause of death. Low serum phosphate was significantly associated with all-cause and infection-related death using time-dependent values (HR, 1.43 [95% confidence interval (CI), 1.06–1.93], *P* = 0.02, and HR, 1.66 [95% CI, 1.02–2.70], *P* = 0.04, respectively). Low serum phosphate was associated with significantly higher infection-related mortality, especially in patients older than 65 years or on dialysis more than one year or with serum albumin lower than 3.9 g/dL (HR, 2.06 [95% CI, 1.13–3.75], *P* = 0.02, HR, 2.19 [95% CI, 1.20–4.01], *P* = 0.01, and HR, 1.77 [95% CI, 1.00–3.13], *P* = 0.05, respectively). Multinomial logistic regression analysis results suggested that low serum albumin, creatinine, and body mass index correlated with low serum phosphate.

**Conclusions:**

Low serum phosphate in dialysis patients was an independent risk factor for infection-related death, especially in elderly patients. Persistently low serum phosphate might be a nutritional biomarker to predict increased susceptibility to infection and in turn worse outcomes in dialysis patients.

## Introduction

The incidence of mortality and morbidity in dialysis patients is a major global concern because the public health burden is consistently high [1]. Independent associations between reduced estimated glomerular filtration rate (eGFR) and the risk of death, cardiovascular events, and hospitalization have been reported [2]. Differences in dialysis mortality are not, however, fully explained by comorbidities or traditional risk factors [2]. Recent speculation has focused on mineral bone disorders (MBDs), including dysregulated hormone balance and altered inflammatory cytokines, as a key factor in the pathogenesis of chronic kidney disease (CKD) [3,4].

High levels of serum calcium, phosphate, calcium–phosphate product, and parathyroid hormone (PTH) are associated with all-cause and cardiovascular-specific mortality in patients with kidney disease [5–8]. In addition, these relationships are observed more clearly when parameters are analyzed as a time-dependent or cumulative time-dependent variable rather than as a baseline value [8]. Vascular calcification, altered cardiac morphological characteristics, and inflammation have been suggested to mediate this relationship [9–14]. However, less effort has gone into assessing the relationship between low levels of serum calcium or phosphate and mortality in dialysis patients because of the rarity of these conditions [7]. Hence, many aspects of the association between mineral metabolism parameters and mortality are still unknown.

The aim of this study was to investigate the prognostic value of serum calcium and phosphate levels relative to mortality in a large prospectively collected national dialysis cohort. Using longitudinal measures of mineral metabolism parameters, we characterized the risk of cause-specific mortality as well as crude mortality associated with different serum calcium and phosphate levels in dialysis patients.

## Materials and methods

### Study cohort

This cohort study was performed using data from the Clinical Research Center for End Stage Renal Disease (CRC for ESRD) in Korea, which is a nationwide, multi-center, prospective observational cohort study (NCT00931970). The study cohort has been described in detail in our previous paper [15]. From July 2009 to June 2011, a total of 3,226 prevalent and incident hemodialysis (HD) or peritoneal dialysis (PD) patients were enrolled from 31 dialysis centers across the country. Eligible patients were at least 20 years old and had measurements of mineral metabolic values at baseline. The CRC registry for ESRD was approved by the medical ethics committees of all participating dialysis centers and informed consent was obtained before inclusion from all patients.

### Ethics statement

The study protocol was approved by Institutional Review Boards of each center before patient enrollment. The names of Institutional Review Boards were as follows. The Catholic University of Korea, Bucheon St. Mary's Hospital; The Catholic University of Korea, Incheon St. Mary's Hospital; The Catholic University of Korea, Seoul St. Mary's Hospital; The Catholic University of Korea, St. Mary's Hospital; The Catholic University of Korea, St. Vincent's Hospital; The Catholic University of Korea, Uijeongbu St. Mary's Hospital; Cheju Halla General Hospital; Chonbuk National University Hospital; Chonnam National University Hospital; Chung-Ang University Medical Center; Chungbuk National University Hospital; Chungnam National University Hospital; Dong A University Medical Center; Ehwa Womens University Medical Center; Fatima Hospital, Daegu; Gachon University Gil Medical Center; Inje University Pusan Paik Hospital; Kyungpook National University Hospital (2011-01-041); Kwandong University College of Medicine, Myongji Hospital; National Health Insurance Corporation Ilsan Hospital; National Medical Center; Pusan National University Hospital; Samsung Medical Center, Seoul; Seoul Metropolitan Government, Seoul National University, Boramae Medical Center; Seoul National University Hospital; Seoul National University, Bundang Hospital; Yeungnam University Medical Center; Yonsei University, Severance Hospital; Yonsei University, Gangnam Severance Hospital; Ulsan University Hospital; Wonju Christian Hospital (in alphabetical order). Contact information for the IRB of Kyungpook National University Hospital is as follows: Tel: +82-53-420-5430, E-mail: knuhmrc@knu.ac.kr. This study was performed in accordance to the 2008 Declaration of Helsinki. Written informed consent was obtained from all patients before inclusion.

### Data collection

**Fig 1:**
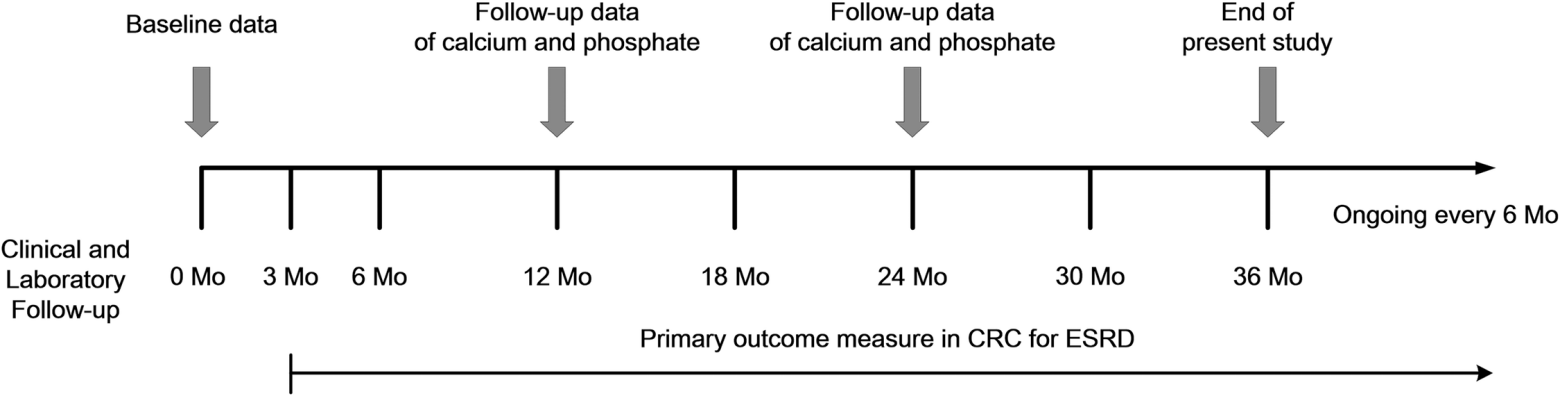
Timeline diagram of the study cohort. The study’s data were derived from patients who were registered on Clinical Research Center for End Stage Renal Disease in Korea July 2009 to June 2011. Mo, month; CRC, Clinical Research Center; ESRD, end-stage renal disease.

Data for patient demographics, primary renal disease, comorbid conditions, and laboratory data were collected at study enrollment. Comorbid conditions and laboratory data were assessed at baseline, 3 months, and 6 months after the initiation of dialysis with subsequent 6-month follow-up examinations (Fig 1). Comorbid conditions included a history of diabetes, congestive heart failure, coronary artery disease, cerebrovascular disease, chronic lung disease, moderate or severe liver disease, and malignancy. The laboratory data set included hemoglobin, serum albumin, blood urea nitrogen, creatinine, eGFR, C-reactive protein (CRP), total cholesterol, ferritin, PTH, calcium, and phosphate levels retrospectively. Mortality was classified as all-cause, cardiovascular, and infection-related. Cardiovascular mortality in patients was defined as death attributable to myocardial infarction, heart failure, arrhythmia, cerebrovascular accident, or sudden death. The primary outcome measure was time to death from any cause. Date and cause of death were reported within 1 month after the event and ascertained by data from Statistics Korea. Patient outcomes were consistently assessed through study period. Patients were censored at the time of kidney transplantation.

### Statistical analysis

Patient characteristics were compared using the Pearson chi-square test or Fisher’s exact test for categorical variables and one-way analysis of variance (ANOVA) with post hoc Scheffe test or Kruskal-Wallis test with Dunn’s post hoc test for continuous variables. Baseline mineral metabolic values were reported according to Kidney Disease Outcomes Quality Initiative (KDOQI) recommended targets for each parameter, as follows: calcium, 8.4 to 9.5 mg/dL (2.10 to 2.37 mmol/L); and phosphate, 3.5 to 5.5 mg/dL (1.13 to 1.78 mmol/L) [16]. We postulated that time-dependent models for evaluation of the relationship between mineral parameters and cause-specific mortality could represent the changes in the clinical condition of a patient before death. We therefore used mineral metabolism parameters obtained at baseline, 12 months, and 24 months during follow-up and evaluated mortality during the year after measuring those values. Hazard ratios (HRs) for all-cause mortality were calculated with Cox proportional hazard model with adjustment. We then applied the proportional hazards model for subdistribution of a competing risk to estimate the subdistribution HRs and 95% confidence intervals (CIs) in relation to primary outcomes. Age, sex, dialysis duration, Modified Charlson Co-morbidity Index (MCCI), CRP, ferritin, use of phosphate binder, and presence of residual renal function (defined as urine volume of more than 100 ml/day) were considered as possible confounders. A multinomial logistic regression model was used to investigate serum phosphate level and nutritional interactions controlling for age, sex, dialysis duration, MCCI, CRP, ferritin, use of phosphate binder, and presence of residual renal function; this model included all variables significantly associated with nutritional markers in multivariate analysis. We performed the analysis based on intention to treat method (not censoring data at the time of a dialysis modality change during the follow-up period). The statistical analysis was performed using SAS for Windows, version 9.2 (SAS Institute Inc., Cary, NC) and R (R Foundation for Statistical Computing, Vienna, Austria; www.r-project.org). *P* values <0.05 were considered statistically significant.

## Results

### Patient characteristics

Our study included 1,998 (61.9%) HD patients and 1,228 (38.1%) PD patients from the CRC for ESRD in Korea. Table 1 describes baseline demographic characteristics and laboratory parameters for the entire cohort and groups separated by baseline phosphate level. Patients had been on dialysis for a mean of 58.2 months and followed for a mean of 19.8 months. The mean age of the patient population was 58.1 years, and 57.1% were men. Body mass indexes (BMIs) were similar in all groups, and diabetes was the most common primary renal disease in all groups. The group with low serum phosphate showed significantly low serum albumin and creatinine levels and significantly high baseline serum calcium and PTH levels compared to the other groups.

**Table 1:** Baseline characteristics and biochemical data in maintenance dialysis patients, stratified by serum phosphate tertile.

		Baseline phosphate (mg/dL)			*P* value
	Total	<3.5	3.5–5.5	>5.5	
	(n = 3,226)	(n = 471)	(n = 1,677)	(n = 1,078)	
Dialysis duration (months)	58.2 ± 52.1	56.5 ± 50.3	58.8 ± 52.0	58.0 ± 53.0	0.680
Follow-up duration (months)	19.8 ± 8.2	19.0 ± 8.2	19.9 ± 8.3	19.9 ± 8.1	0.099
Age (y)	58.1 ± 13.4	63.3 ± 12.7^a^	59.4 ± 13.0^b^	53.8 ± 12.9^c^	<0.001
Sex (% male)	1,842 (57.1)	255 (54.1)	958 (57.1)	629 (58.3)	0.306
BMI (kg/m^2^)	22.8 ± 3.4	22.1 ± 3.2^a^	22.7 ± 3.3^b^	23.2 ± 3.6^c^	<0.001
Primary renal disease, n (%)					
Diabetes	1,392 (49.1)	208 (51.6)	781 (52.5)	403 (42.7)	<0.001
Hypertension	653 (23.0)	91 (22.6)	332 (22.3)	230(24.4)	
Glomerulonephritis	515 (18.2)	50 (12.4)	260 (17.5)	205(21.7)	
Others	275 (9.7)	54 (13.4)	116 (7.8)	105 (11.1)	
Comorbid conditions, n (%)					
Diabetes	1,177 (47.1)	177 (51.8)	649 (50.7)	351 (40.0)	<0.001
Congestive heart failure	252 (10.1)	36 (10.6)	136 (10.6)	80 (9.1)	0.496
Coronary artery disease	272 (10.9)	47 (13.9)	151 (11.8)	74 (8.5)	0.009
Peripheral vascular disease	115 (4.6)	20 (5.9)	66 (5.2)	29 (3.3)	0.066
Arrhythmia	43 (1.7)	4 (1.2)	22 (1.7)	17 (1.9)	0.656
Cerebrovascular disease	206 (8.3)	38 (11.1)	109 (8.5)	59 (6.8)	0.040
Chronic lung disease	176 (7.1)	35 (10.3)	89 (7.0)	52 (5.9)	0.030
Peptic ulcer disease	145 (5.8)	26 (7.6)	80 (6.3)	39 (4.5)	0.064
Moderate or severe liver disease	76 (3.0)	19 (5.6)	34 (2.7)	23 (2.6)	0.014
Connective tissue disease	212 (8.5)	27 (7.9)	111 (8.7)	74 (8.5)	0.906
Malignancy	112 (4.5)	27 (7.9)	55 (4.3)	30 (3.4)	0.003
Smokers, n (%)					
Non-smoker	1,935 (61.6)	292 (63.8)	1,018 (62.1)	625 (59.8)	<0.001
Smoker	318 (10.1)	31 (6.8)	140 (8.5)	147 (14.1)	
Ex-smoker	890 (28.3)	135 (29.5)	481 (29.3)	274 (26.2)	
Residual urine volume, n (%)					
>100 ml/day	1,200 (32.2)	160 (34.0)	658 (39.2)	382 (35.4)	0.084
≤100 ml/day	2,026 (62.8)	311 (66.0)	1,019 (60.8)	696 (64.5)	
Systolic pressure (mmHg)	138.6 ± 21.2	136.1 ± 20.6^a^	138.6 ± 20.8^b^	139.8 ± 21.9^b^	0.010
Diastolic pressure (mmHg)	78.5 ± 12.7	76.9 ± 11.6^a^	78.1 ± 12.7^a^	79.7 ± 13.2^b^	<0.001
Laboratory data					
Hemoglobin (g/dL)	10.1 ± 1.7	10.1 ± 1.6^a^	10.2 ± 1.6^a^	9.9 ± 1.8^b^	<0.001
Albumin (g/dL)	3.7 ± 0.5	3.6 ± 0.5^a^	3.7 ± 0.5^b^	3.7 ± 0.6^b^	<0.001
Blood urea nitrogen (mg/dL)	64.8 ± 27.3	49.1 ± 26.8^a^	60.3 ± 23.3^b^	78.8 ± 29.6^b^	<0.001
Creatinine (mg/dL)	9.6 ± 3.8	7.6 ± 3.7^a^	8.9 ± 3.4^b^	11.5 ± 3.8^c^	<0.001
Baseline eGFR (ml/min/1.73 m^2^)	6.1 ± 4.0	8.1 ± 6.3^a^	6.4 ± 3.6^b^	4.7 ± 2.3^c^	<0.001
CRP (mg/dL)	0.9(0.1,10.1)	1.5(0.2,10.1)^a^	0.8(0.1,10.1)^b^	1.1(0.2,10.1)^b^	0.007
Total cholesterol (mg/dL)	162.4 ± 42.2	158.9 ± 40.7	162.1 ± 40.6	164.6 ± 45.4	0.056
Triglycerides (mg/dL)	131.8 ± 89.3	129.7 ± 77.2	129.4 ± 87.4	136.7 ± 97.0	0.148
LDL (mg/dL)	91.1 ± 34.0	88.8 ± 35.4	91.0 ± 32.9	92.4 ± 34.9	0.229
Ferritin (ng/dL)	194.0 (96.5,370.2)	228.0 (120.0,464.7)^a^	194.1 (100.5,359.3)^b^	176.8 (88.4,355.5)^b^	<0.001
Baseline calcium (mg/dL)	8.8 ± 1.0	9.0 ± 0.9^a^	8.8 ± 0.9^b^	8.6 ± 1.1^c^	<0.001
Baseline PTH (mg/dL)	238.6 ± 262.5	162.6 ± 199.7^a^	222.5 ± 246.1^b^	299.4 ± 298.3^c^	<0.001

Values are shown as mean ± standard deviation or median (interquartile range). The different superscripts denote significant differences between groups not sharing the same superscript at 0.05 level based on Scheffe test or Dunn’s post hoc test.

BMI, body mass index; eGFR, estimated glomerular filtration rate; CRP, C-reactive protein; LDL, low-density lipoprotein; PTH, parathyroid hormone.

### Cause of death

**Fig 2:**
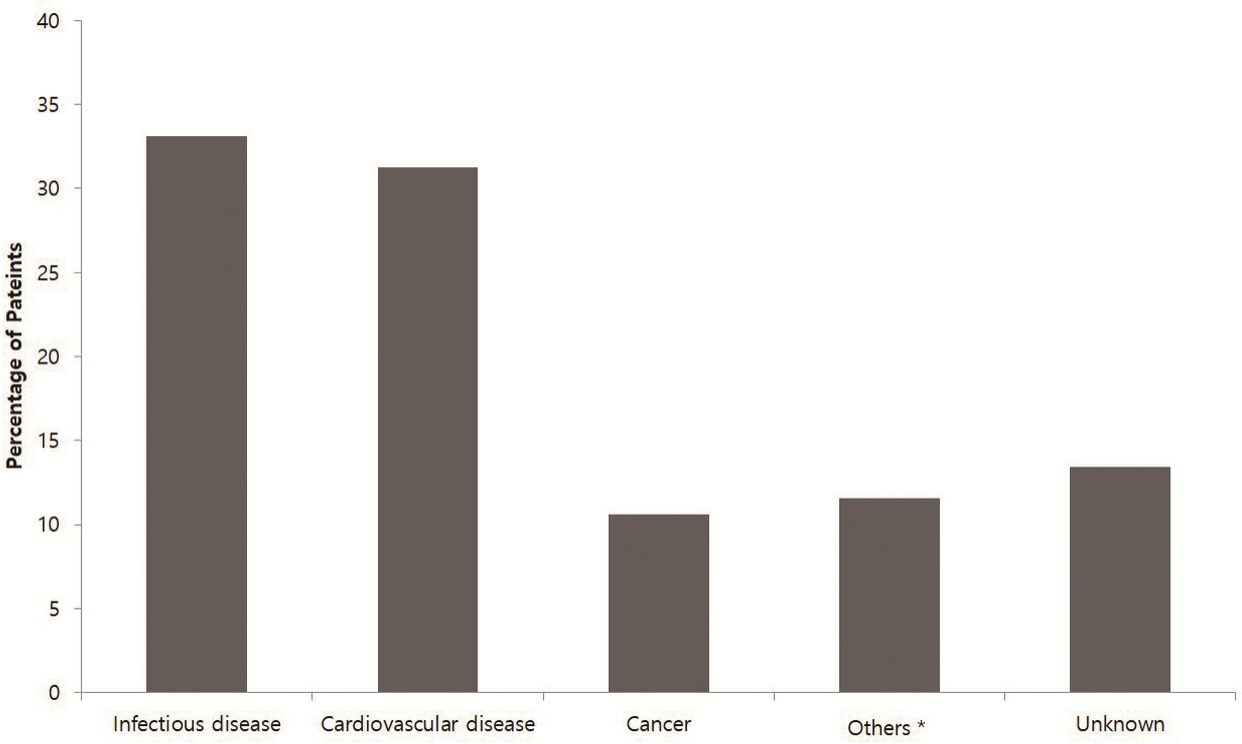
Causes of death in study cohort. * Others included liver disease, gastrointestinal disease, endocrine or hematologic disease, chronic obstructive pulmonary disease, and suicide.

There were 320 deaths during study period: 195 (61%) in the HD group and 125 (39%) in the PD group (Fig 2). Infectious disease was the most common cause of death in the cohort and occurred in 106 patients (33.1%). Cardiovascular disease was the second most common cause of death, documented in 100 patients (31.3%), followed by deaths with unknown causes in 43 patients (13.4%) and cancer in 34 patients (10.6%).

### Association of serum calcium and phosphate with mortality

A Cox proportional hazards model and proportional hazards model for subdistribution of a competing risk were used to calculate adjusted HRs for all-cause and cause-specific mortality in categories of patients who had serum calcium or phosphate levels less than, within, or greater than the recommended targets (Table 2). A low serum phosphate level showed a significant association with all-cause mortality and infection-related mortality (HR, 1.43 [95% confidence interval (CI), 1.06–1.93], *P* = 0.02, and HR, 1.66 [95% CI 1.02–2.70], *P* = 0.04, respectively). However, serum calcium was not significantly associated with any kind of mortality in this model.

**Table 2:** Associations of serum calcium and phosphate at different time points with mortality (baseline, 12 months, 24 months).

	All-cause		Subdistribution Hazard Model			
			Cardiovascular		Infection-related	
	Adjusted HR (95% CI)	*P* value	Adjusted HR (95% CI)	*P* value	Adjusted HR (95% CI)	*P* value
Serum calcium (mg/dL) tertile: level						
<8.4	0.93 (0.68–1.29)	0.668	1.22 (0.56–2.69)	0.620	0.81 (0.44–1.47)	0.480
8.4–9.5	Reference		Reference		Reference	
>9.5	1.10 (0.82–1.48)	0.527	0.91 (0.39–2.14)	0.830	0.97 (0.55–1.70)	0.920
Serum phosphate (mg/dL) tertile: level						
<3.5	1.43 (1.06–1.93)	0.018	0.94 (0.38–2.33)	0.890	1.66 (1.02–2.70)	0.041
3.5–5.5	Reference		Reference		Reference	
>5.5	1.16 (0.86–1.57)	0.337	1.24 (0.55–2.78)	0.600	0.91 (0.52–1.62)	0.750

Adjusted for age, sex, dialysis duration, Modified Charlson Co-morbidity Index, C-reactive protein level, ferritin level, use of phosphate binder, and presence of residual renal function.

HR, hazard ratio; CI, confidence interval.

### Subgroup analysis

**Fig 3:**
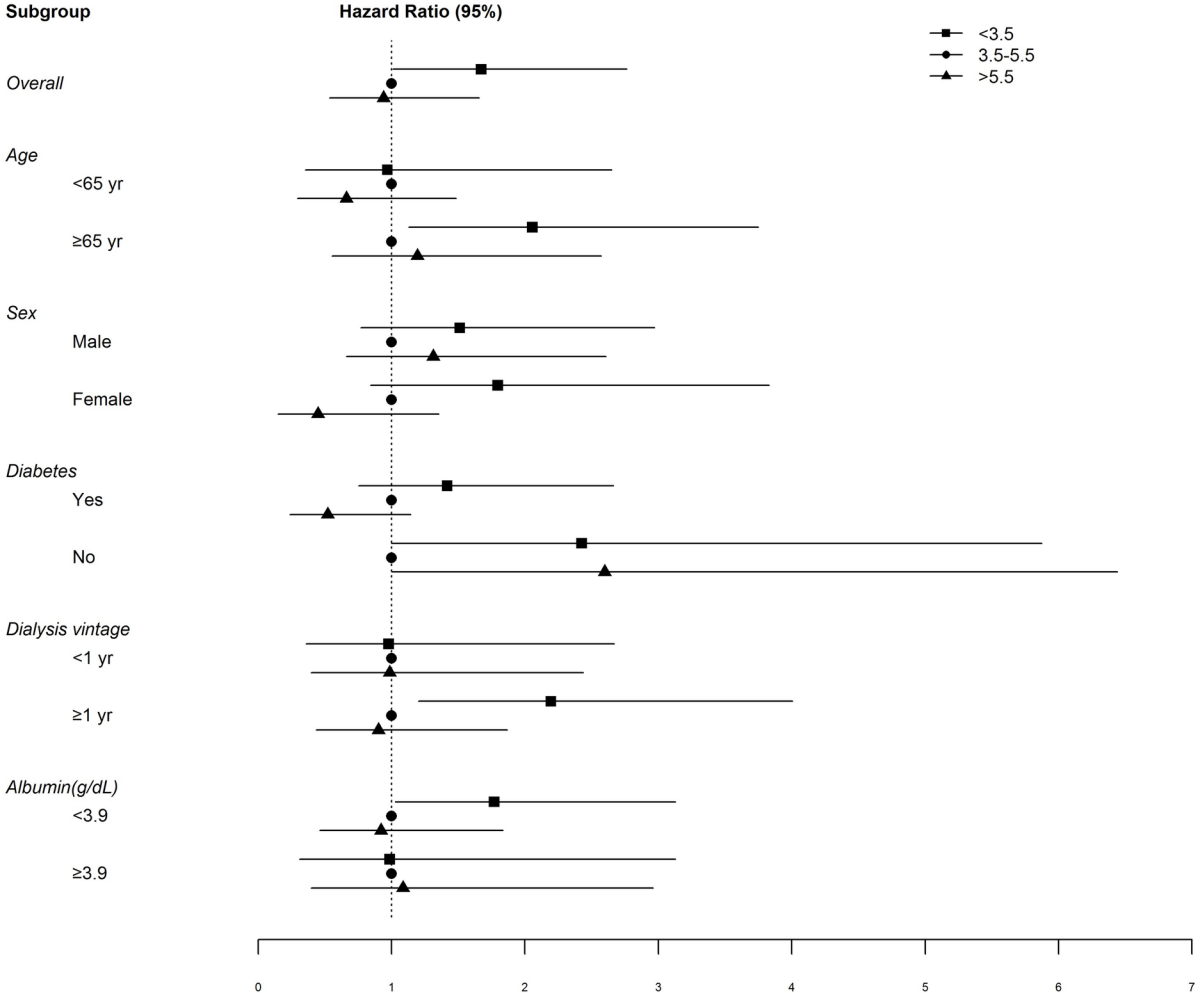
Subgroup analysis of infection-related mortality at different time points. Adjusted for age, sex, dialysis duration, Modified Charlson Co-morbidity Index, C-reactive protein level, ferritin level, use of phosphate binder, and presence of residual renal function.

Fig 3 shows the forest plot of the results of the subgroup analysis of infection-related mortality at different time points. This analysis revealed that the prognostic value of a low serum phosphate level was significant in high-risk groups such as patients age ≥65 years or on dialysis one year or longer, or with serum albumin <3.9 g/dL. Other subgroups divided by sex, and underlying diabetes showed no significant infection-related mortality relative to different serum phosphate levels.

### Association between serum phosphate level and nutritional markers

The result of multinomial logistic regression analysis with serum phosphate level as the dependent variable showed that serum albumin, creatinine, and BMI were independently associated with low serum phosphate level in both crude and adjusted models (Table 3). Patients who had higher serum albumin had a 32% lower risk of having a low phosphate level at an interval of 1 g/dL in a model adjusted for other nutritional markers. Additionally, higher serum creatinine and higher BMI were also related to a lower risk of low serum phosphate (5.4% and 4.4% per each unit, respectively). However, a higher total cholesterol level was not associated with low serum phosphate.

**Table 3:** Crude and adjusted ORs and 95% CIs for phosphate levels according to nutritional markers.

	Crude OR (95% CI)		Adjusted OR (95% CI)	
Phosphate (mg/dL)	<3.5	>5.5	<3.5	>5.5
Serum albumin	0.661 (0.551–0.794)	1.001 (0.865–1.160)	0.682 (0.539–0.863)	0.789 (0.653–0.952)
Creatinine	0.902 (0.870–0.934)	1.208 (1.178–1.240)	0.946 (0.904–0.991)	1.102 (1.068–1.137)
Total cholesterol	1.000 (0.997–1.002)	1.002 (1.000–1.004)	1.001 (0.998–1.004)	1.001 (0.999–1.004)
BMI	0.954 (0.924–0.985)	1.055 (1.031–1.080)	0.956 (0.921–0.993)	1.060 (1.031–1.090)

Reference serum phosphate level was between 3.5 to 5.5 mg/dL. Adjusted for age, sex, dialysis duration, Modified Charlson Co-morbidity Index, C-reactive protein, ferritin, albumin, use of phosphate binder, and presence of residual renal function.

OR, odds ratio; CI, confidence interval; BMI, body mass index.

## Discussion

Several studies have evaluated MBD in CKD or ESRD, and many clinicians are attempting to achieve the KDOQI-MBD target in their patients. However, only a few publications have reported the relationship between low serum phosphate and crude mortality with cause-specific mortality such as that related to infection. According to several previous cohort studies, infection was the second most common cause of death following cardiovascular disease in patients with ESRD [17,18]. The rates of hospitalization due to infection are increasing [1], and mortality secondary to sepsis is approximately 50-fold higher in dialysis patients compared with the general population [19].

In the current study, we confirmed that low serum phosphate is significantly associated with increased all-cause mortality and infection-related mortality in dialysis patients. Of note, we found that low serum phosphate levels combined with being age 65 years or older or with dialysis vintage longer than one year or with a serum albumin level lower than 3.9 g/dL were related to a significantly increased infection-related mortality.

The occurrence of hypophosphatemia in dialysis patients is caused by avoidance of phosphate-containing food (milk, meat, fish, eggs, etc.), malnourishment, parenteral nutrition, and use of medications such as phosphate-binding antacids or erythropoietin [20,21]. Some studies have already reported strong relations between hyperphosphatemia or hypercalcemia and elevated mortality among ESRD patients [5,22]. One effect of these conditions is calcification of the systemic vasculature, including the coronary arteries, and the calcification has been associated with increased arterial stiffness and mortality [23]. However, hypophosphatemia is considered less important than hyperphosphatemia or hypercalcemia and remains poorly studied. Cardiovascular death is the most common cause of death in western ESRD patients, but few patients have hypophosphatemia—about 8% in various cohorts [24–26]—so it is hard to evaluate cause of death in this minor population.

In this cohort study, the most common cause of death in dialysis patients was infection-related. This result is different from western studies in which cardiovascular death was reported to be a leading cause of death in ESRD patients [17,18]. We have already confirmed in our previous study that infection was the most common cause of death in our Korean ESRD cohort [15], which we have verified again here. Cardiovascular death was the second most common cause of death in this Korean cohort, a difference from western groups that might be related to a relatively low obesity rate and low percentage of comorbid diseases such as congestive heart failure, coronary artery disease, and peripheral vascular disease. The obesity rate among Korean adults is very low compared to other Organization for Economic Co-operation and Development (OECD) countries, according to a 2014 OECD report (Korea 4.6%, United States 35.3%, Australia 28.3%, and New Zealand 31.3%) [27]. In addition, in our cohort, the rate of causable comorbidities, such as congestive heart failure, coronary artery disease, and peripheral vascular disease, was much lower than in the US population [17] or ANZDATA registry [18]. Furthermore, the percentage of hypophosphatemia was almost 15% in this Korean cohort group, which was nearly double compared with other western studies [24–26]. We speculated that a Korean diet and use of drugs might affect this result, so we sought to evaluate the relations between hypophosphatemia and infection-related mortality more precisely.

We were able to show that low serum phosphate in dialysis patients was associated with all-cause mortality and infection-related mortality. The main mechanisms underlying the association are not yet known, however. We focused on that phosphate might also be a nutritional biomarker, and low phosphate could represent poor nutritional state as demonstrated in previous studies [28,29]. Furthermore, there is a possibility that this poor nutritional state is associated with susceptibility to infection. The result of multinomial logistic regression analysis supported our hypothesis. There were close relations between low serum phosphate level and other nutritional biomarkers, such as serum albumin, creatinine, and BMI. Serum albumin is the most commonly used and reliable nutritional marker in CKD and ESRD patients, and protein-rich foods are the main source of dietary phosphate [30]. Serum creatinine is a useful biochemical marker for assessing nutritional status in maintenance dialysis patients, as well [31]. Therefore, a low phosphate level is considered to represent poor nutritional status.

Also, in this study, serum total cholesterol level was not related with serum phosphate level. Though previous studies have reported it correlated with nutritional status in chronic dialysis patients, the former might be a less sensitive nutritional biochemical marker [31,32]. Generally, it is rare for decreased intake alone to cause hypophosphatemia [22], but malabsorption and use of specific drugs, such as phosphate-binding antacids or erythropoietin, can decrease intestinal phosphate absorption or shift phosphate from the extracellular space into the cell [21]. Neutrophil dysfunction caused by hypophosphatemia is another possible explanation for this result. Hypophosphatemia can reduce the ATP content of leukocytes and attenuate neutrophil phagocytosis, intracellular killing, consumption of oxygen, and generation of superoxide during phagocytosis [22]. As a result, susceptibility to infection could be increased.

In the subgroup analysis, low phosphate in patients aged 65 years or older or on dialysis more than one year or with a serum albumin level less than 3.9 mg/dL was significantly associated with infection-related mortality. Hence, we should attend more to controlling serum phosphate level in this high-risk group.

The strength of our analysis is that we used a large prospective cohort of patients with established kidney failure on both HD and PD therapy from CRC for ESRD in Korea. In addition, we introduced a time-dependent model to demonstrate association regarding time. Recent cohort studies have shown conflicting results. Rivara *et al*. [33] reported no relationship between low serum phosphate, and all-cause and cause-specific mortality in a US ESRD population. Another large cohort study undertaken in Asia, however, reported that low serum phosphate level was associated with high all-cause mortality in dialysis patients [34]. Nevertheless, a relationship between low serum phosphate level and cause-specific mortality including infection-related mortality was not established. To our best knowledge, this is the first large-scale study based on a prospective cohort to investigate the relations between low serum phosphate level and infection-related mortality in dialysis patients.

There are some limitations in this study. First, this is an observational study, thus it is difficult to clarify the causal relationship between variables. Second, for the purpose of improving generalizability of the result, we included both incident and prevalent dialysis patients in study cohort. There, however, is a possibility of selection bias. To control this, all models were adjusted for dialysis vintage in study population and time dependent models were applied. We did not find any significant association between serum calcium level and any type of mortality. This result might be related to the fact that the number of patients in each classified group was relatively low and the follow-up duration was short in this cohort study. Larger and long-term follow-up studies are deemed necessary in the near future.

In conclusion, a low serum phosphate level in dialysis patients was significantly associated with high infection-related mortality. In particular, we found a stronger association in older patients or long-term dialysis patients or patients who had low serum albumin. A low phosphate level is considered to be a nutritional biomarker because of its close relationship with other nutritional markers, such as serum albumin, creatinine level, and BMI. Therefore, these results suggest that it seems reasonable for ESRD patients to adequate phosphate level, and it might be helpful to prevent worse outcomes. Clinicians should pay more attention to patient nutritional status.
